# Comparisons of outcomes of open surgery versus endovascular intervention for thrombotic popliteal artery aneurysm with acute lower limb ischemia: a systematic review

**DOI:** 10.1186/s12893-022-01843-1

**Published:** 2022-11-18

**Authors:** Xixi Xiao, Ruijia Feng, Mingshan Wang, Hanqi Sun, Tianzhen Jing, Lianhua Su, You Fan, Zuojun Hu, Shenming Wang, Siwen Wang

**Affiliations:** 1 Department of Vascular Surgery, National-Guangdong Joint Engineering Laboratory for Diagnosis and Treatment of Vascular Diseases, The First Affiliated Hospital, Sun Yat-Sen University, No.58, Zhongshan 2nd road, Yuexiu District, Guangzhou, 510080 Guangdong China; 2 Zhongshan School of Medicine, Sun Yat-Sen University, Guangzhou, 510080 China

**Keywords:** Thrombotic popliteal artery aneurysm, Acute lower limb ischemia, Open surgery, Endovascular intervention

## Abstract

**Background:**

Thrombotic popliteal artery aneurysm (PAA) with acute lower limb ischemia (ALI) is a serious disease leading to amputation. The choice of emergency procedures is not clearly defined, and the difference in therapeutic efficiency between open surgery and endovascular intervention is still unclear.

**Method:**

We conducted a comprehensive search through PubMed, Wiley Online Library and ScienceDirect. According to the predefined inclusion and exclusion criteria, eligible articles were screened out, and all relevant data were extracted for further analysis. Our study was designed and developed based on Preferred Reporting Items for Systematic Reviews and Meta-Analyses (PRISMA) Guideline. We critically assessed all included articles by Joanna Briggs Institute (JBI) Critical Appraisal Checklists and the Methodological Index for Non-Randomized Studies (MINORS).

**Result:**

A total of 29 articles (1338 patients/1387 limbs) were included in the study. After a 1-year follow-up, the primary patency rate of the open surgery group was significantly lower than that of the endovascular intervention group (72.65 vs. 81.46%, P = 0.004), but without significant difference in the secondary patency rate (86.19 vs. 86.86%, P = 0.825). The limb salvage rate of the open surgery group was also significantly lower (83.07 vs. 98.25%, P < 0.001). After the 2-year follow-up, the primary patency rate of the open surgery group was still significantly lower (48.57 vs. 59.90%, P = 0.021).

**Conclusion:**

The outcome of endovascular intervention was better than that of open surgery especially in the 1-year limb salvage rate and primary patency rate at the 1-year and 2-year follow-ups.

## Introduction

Popliteal artery aneurysm (PAA) is the most common peripheral artery aneurysm. Patients usually have no obvious symptoms, except for those who suffer sudden acute lower limb ischemia (ALI), which may lead to numbness, pain, intermittent claudication, acute ischemic necrosis of the lower limbs or even amputation [1]. Approximately 17–46% of patients with PAA may experience severe ALI [2–5], and emergency surgery must be considered for treatment. Even though emergency procedures were conducted, the amputation rate was still very high, and the postoperative patency rate was lower than that of chronic ischemia with PAA due to the lack of clarity in selecting appropriate surgical procedures [6]. Because of the limited epidemiological incidence and minimal studies, defined surgical treatment guidelines are not available, and experienced vascular surgeons should assist in making decisions.

Involving case reports and retrospective case series studies with small sample sizes, the existing studies on surgical procedures mainly focused on the therapeutic efficiency of preoperative and intraoperative thrombolysis. The results indicated that preoperative and intraoperative thrombolysis increased the long-term patency rates [6–8]. However, randomized controlled trials to evaluate better surgical procedures have not been conducted. Therefore, we aimed to evaluate the therapeutic efficiency of open surgery and endovascular intervention through a systematic review of the included articles to provide evidence of appropriate surgical strategies for treating thrombotic PAA with ALI.

## Materials and methods

### Data sources and inclusion criteria

**Fig. 1:**
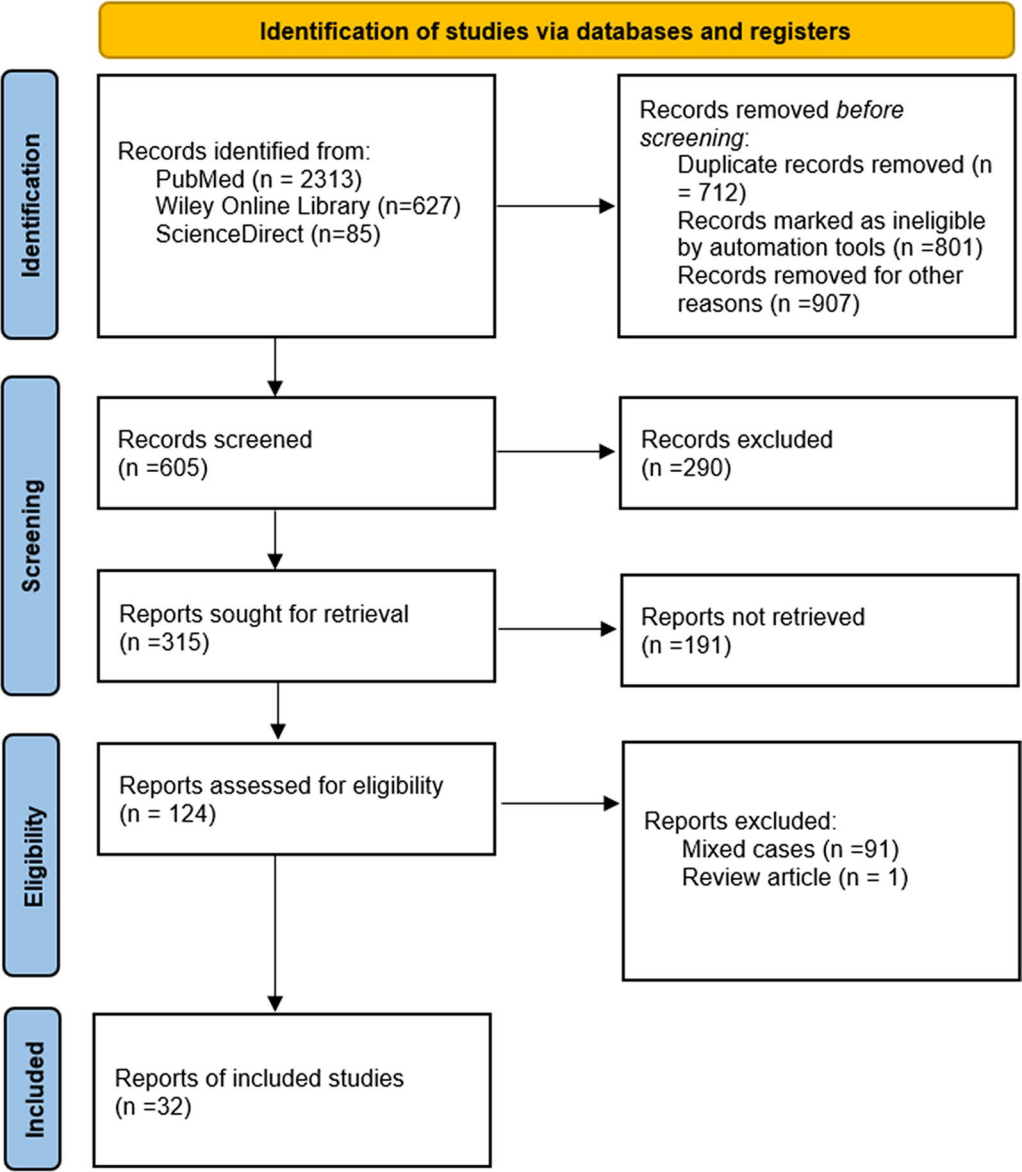
Flow diagram of the included studies based on the Preferred Reporting Items for Systematic Review and Meta-Analysis (PRISMA)

We conducted a comprehensive search in PUBMED, Wiley Online Library and ScienceDirect using the keyword combination "acute ischemia" or "acute embolism" and "popliteal arterial aneurysm" and "thrombosis". We also searched conference abstracts and the references of the studies identified. All articles about thrombotic PAA with ALI (number of cases ≥ 1) published between January 2000 and December 2021 in English were identified. The latest search date was January 5, 2022. Two authors independently screened titles and abstracts to determine potential eligibility for analysis. When discrepancies occurred, consensus was achieved after further discussion. The study retrieval and inclusion process are shown in Fig. 1 according to Preferred Reporting Items for Systematic Reviews and Meta-Analyses (PRISMA) Guideline. At last, the included studies were case reports, case series studies and retrospective nonrandomized case–control studies.

The inclusion and exclusion criteria were as follows: (1) articles about PAA with thrombosis in aneurysm sac and ALI; (2) included data on the following four variables: patient characteristics, surgical procedures, perioperative complications and follow-up data; (3) if multiple case series were reported from the same center, only the latest published paper or the paper containing the most detailed information was included; (4) if the same center reported different cases in different years, the data were analyzed separately; and (5) sufficient data (≥ 20% of predefined variables were available) were needed.

### Data extraction and definition

Two authors independently extracted the following data from the selected articles: first author, year of publication, predefined variables included the patient characteristics, surgical procedures, perioperative complications, and follow-up data. All original data were filled into a standard data-extraction template. Unspecified information was recorded as a missing value (N.A.), so the overall number of patients analyzed for different variables might be different. The patient characteristics included sex, age, smoking, diabetes, hypertension, hyperlipidemia, respiratory diseases, renal diseases, atrial fibrillation, etc. Respiratory diseases included chronic obstructive pulmonary disease (COPD), emphysema, pulmonary embolism, etc. Renal diseases included renal insufficiency, renal failure, partial renal infarction, etc. Mild ALI cases defined the patients with symptoms and signs included pain, pulselessness, pallor, paresthesia, paralysis (5P) and low skin temperature. Severe ALI symptoms and signs included skin color change (dusky blue) and gangrene at the distal lower limb. The surgical procedures can be separated into open surgery and endovascular intervention. Open surgery involved proximal and distal PAA ligation with graft bypass surgery and PAA resection with in situ graft replacement (artificial blood vessel or great saphenous vein was adopted as the graft). Endovascular intervention included catheter thrombolysis, thrombectomy involved thromboaspiration devices (AngioJet and Indigo), and covered stent graft implantation (or a combination with thrombolysis and/or thrombectomy). Most of stent graft were Viabahn and Hemobahn. The type of drugs used when thrombolysis included alteplase (also called rt-PA) and urokinase. If a patient got the hybrid treatment like open surgery right after endovascular intervention, the patient was also included in open surgery group. Most surgical procedures were performed under general anesthesia. Local anesthesia was applied only in some cases with endovascular intervention. In graft bypass surgery, the proximal and distal ends of PAA were ligated after ideal exposure, and then the blood flow was reconstructed with artificial blood vessels or great saphenous vein grafts. For in situ graft replacement, PAAs were opened or resected after controlling the proximal and distal ends, and the popliteal artery was replaced with an in situ graft, which was an artificial blood vessel or great saphenous vein graft, followed by wrapping by the preserved aneurysm wall. Covered stent graft implantation was performed to exclude PAA. For thrombolysis or thrombectomy, the surgeon usually made an access at the femoral artery on the same side and inserted an aspiration thrombectomy catheter or a thrombolysis catheter to recanalize the blood flow in the ischemic lower limbs. Computed tomography of the artery (CTA) was performed before the operation to determine the lesion segments and intraluminal blood flow of PAA in most cases. CTA was also applied to evaluate the outflow tract below the knee and the feasibility of endovascular repair. Patients who underwent covered stent graft implantation needed to undergo angiography again to assess aneurysm cavity exclusion and distal blood flow (at least one outflow tract patent). The technical success referred to the relief of ALI, successful management of PAA (exclusion by stent graft, resection or isolation) and rehabilitation meeting the discharge requirements. The follow-up period was defined as the time from discharge to the longest time point reported in follow-up cases. The follow-up information included the relief of ALI, 1-year and 2-year primary patency rates, secondary patency rates, survival rates and limb salvage rates. Primary patency was defined as the duration of time absence of any stenosis or obstruction without re-intervention, while secondary patency was defined as the total patency with secondary intervention. We contacted the authors of the included articles if essential information was unclear or missing.

### Quality assessment

To figure out whether the selected studies were appropriate for inclusion in the systemic review. Two authors independently assessed all included articles by the Joanna Briggs Institute (JBI) Critical Appraisal Checklist for Case Reports, JBI Critical Appraisal Checklist for Case Series [9] and the Methodological Index for Non-Randomized Studies (MINORS) [10]. JBI checklists contained a total of 8 and 10 questions respectively. Every question was given “yes”, “no”, “unclear” or “not applicable”. MINORS involved 12 items, which were scored as 0 (not reported), 1 (reported but inadequate), or 2 (reported and adequate). No discrepancies existed between two authors.

### Outcome measure

In the systemic review, the primary outcomes were comparisons of the follow-up results included the relief of ALI, 1-year and 2-year primary patency rates, secondary patency rates, survival rates and limb salvage rates. The secondary outcomes were comparisons of comorbidities, peri-operative conditions, and technical success rates between patients in the open surgery group and the endovascular intervention group.

### Statistical analysis

We used SPSS V26 for statistical analysis. The ratio of events was calculated as the number of events divided by the overall number of cases included in the analysis of the variable. The results are presented as the mean ± standard deviation (SD) or median and range (quartile). Bilateral χ ^2^ test or Fisher’s exact value test was used to compare categorical variables, and the t test or Mann–Whitney U test was used to compare continuous variables. Kaplan–Meier survival analysis was used to compare the survival rate during follow-up. P < 0.05 was defined as statistically significant.

## Results

### Overview of literature sources and quality assessment of included papers

**Fig. 2:**
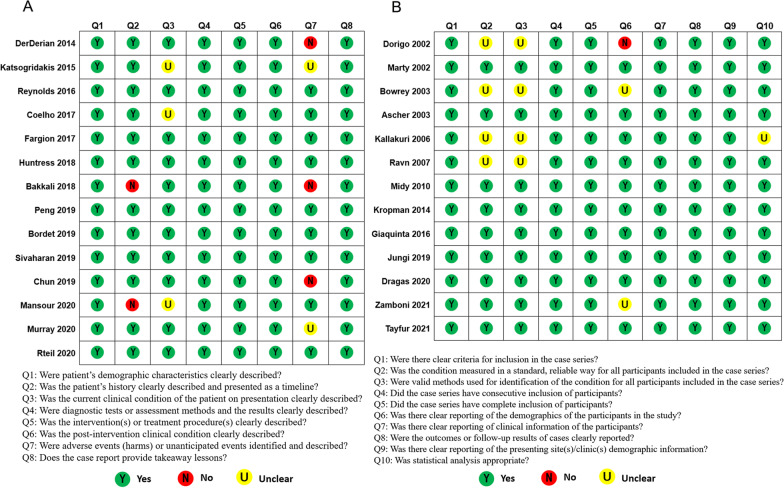
The quality evaluation of 14 case reports by the Joanna Briggs Institute (JBI) Critical Appraisal Checklist for Case Reports **A** and 13 case series by JBI Critical Appraisal Checklist for Case Series (**B**)

A total of 29 English articles (1338 patients/1387 limbs) about thrombotic PAA with ALI were included in this review [6–8, 11–36] (Table 1). Among them, 14 articles are case reports, 13 articles are case series studies, and 2 articles are nonrandomized comparative studies. The MINORS scores of 2 nonrandomized comparative studies were 20 and 20.5 (scores range: 0–24). According to the assessment results of JBI Checklists and the MINORS scores, all articles are considered to be of high quality (Fig. 2). Because some data cannot be available in some articles even though we contacted the authors of articles, there may be biased results when analysis including these studies (Tables 1 and 2).

**Table 1:** Articles included in the study

Authors	Year	Case	Limbs	Operation	Mean or median age (years)	Range of age (years)	Mean or median follow-up period (months)	Range of follow-up period (months)
Dorigo et al. [11]	2002	89	109	Open	66	28–91	26	1–120
Marty et al. [12]	2002	13	19	Open&Endovascular	76	NA	15	NA
Bowrey et al. [13]	2003	46	47	Open	73	43–94	35	1–119
Ascher et al. [14]	2003	25	34	Open	74	NA	13	1–48
Kallakuri et al. [15]	2006	10	10	Open	76.2	51–99	15.6	NA
Ravn et al. [16]	2007	229	235	212Open	NA	31–94	NA	NA
Midy et al. [17]	2010	50	57	Endovascular	72	57–96	36	6–96
Pulli et al. [18]	2013	312	312	Open&Endovascular	72.1	NA	30.5	1–156
DerDerian et al. [19]	2014	1	1	Endovascular	68	68	NA	NA
Kropman et al. [20]	2014	202	202	Open&Endovascular	66.4	NA	NA	1–183
Katsogridakis et al. [21]	2015	1	1	Endovascular	37	37	1.5	1.5
Giaquinta et al. [22]	2016	11	11	Endovascular	74	NA	24	NA
Reynolds et al. [23]	2016	1	1	Endovascular	72	72	6	6
Coelho et al. [24]	2017	1	1	Open	54	54	NA	NA
Fargion et al. [25]	2017	6	6	Endovascular	76.5	NA	28.6	NA
Huntress et al. [26]	2018	1	1	Open	64	64	12	12
Bakkali et al. [27]	2018	1	1	Open	20	20	3	3
Peng et al. [28]	2019	1	1	Open	59	59	41	41
Bordet et al. [29]	2019	1	1	Open	84	84	12	12
Sivaharan et al. [30]	2019	1	1	Open	6	6	72	72
Jungi et al. [7]	2019	51	51	Open	75	46–97	41	4–114
Chung et al. [31]	2019	1	1	Open	58	58	NA	NA
Mansour et al. [8]	2020	2	2	Open	63.5	51–76	7	6–8
Murray et al. [32]	2020	1	1	Open	26	26	2	2
Dragas et al. [6]	2020	156	156	Open&Endovascular	71.2	NA	55	NA
Wrede et al. [33]	2020	39	39	Open&Endovascular	NA	42–90	NA	0.03–128
Rteil et al. [34]	2020	1	1	Endovascular	75	75	12	12
Zamboni et al. [35]	2021	22	22	Open	72.4	NA	31.5	18.9–44.1
Tayfur et al. [36]	2021	63	63	Endovascular	76.35	NA	46.05	NA

**Table 2:** Baseline patient characteristics of the open surgery group and the endovascular intervention group

Variables	Overall	Open surgery	Endovascular intervention	P value
Gender(male)	72.50% (970/1338)	73.11% (560/766)	54.58% (167/306)	0.000
Smoking	60.95% (562/922)	63.07% (234/371)	72.48% (216/298)	0.010
Diabetes	21.44% (199/928)	27.84% (103/370)	20.33% (62/305)	0.024
Hypertension	70.72% (657/929)	79.57% (296/372)	73.03% (222/304)	0.046
Hyperlipidemia	45.20% (381/843)	51.74% (164/317)	42.49% (116/273)	0.025
Respiratory diseases	28.32% (162/572)	29.67% (100/337)	26.38% (62/235)	0.390
Renal diseases	24.23% (71/293)	28.80% (55/191)	21.57% (11/51)	0.303
Atrial fibrillation	39.25% (73/186)	42.65% (58/136)	30.00% (15/50)	0.117
Heart failure	26.83% (55/205)	27.71% (46/166)	23.08% (9/39)	0.689
Myocardial infarction	36.22% (318/878)	39.41% (134/340)	33.33% (95/285)	0.116
Mild ALI cases	45.51% (421/925)	38.82% (165/431)	56.51% (165/292)	0.000
At least one of runoff	60.59% (661/1091)	53.96% (341/632)	52.10% (124/238)	0.625
None of runoff	38.68% (422/1091)	46.04% (291/632)	47.06% (112/238)	0.789
Technical success	90.73% (988/1089)	91.71% (520/567)	90.80% (227/250)	0.668
Follow up	97.92% (1083/1106)	97.38% (521/535)	97.70% (298/305)	0.774
Duration(month)	1–183	1–156	1–124	–
Perioperative death	0.80% (7/874)	0.88% (5/569)	0.32% (2/305)	1.000
Popliteal artery related	0.34% (3/874)	0.53% (3/569)	0.00% (0/305)	0.556
Non-popliteal artery related	0.46% (4/874)	0.35% (2/569)	0.66% (2/305)	0.614
1-year follow-up death	3.35% (29/865)	1.07% (6/563)	7.62% (23/302)	0.000
Popliteal artery related	0.35% (3/865)	0.53% (3/563)	0.00% (0/615)	0.556
Non-popliteal artery related	3.01% (26/865)	0.53% (3/563)	7.62% (23/615)	0.000
2-year follow-up death	6.55% (27/412)	3.81% (8/210)	9.41% (19/202)	0.028
Popliteal artery related	0.73% (3/412)	1.43% (3/210)	0.00% (0/515)	0.248
Non-popliteal artery related	5.83% (24/412)	2.38% (5/210)	9.41% (19/515)	0.003

### Characteristics of included patients

The patient characteristics are shown in Table 2. The male percentage was 72.50% (970/1338). The percentage of patients with hypertension was 70.72% (657/929), and more than half of the patients were smokers (60.95%). Other complications included respiratory diseases (28.32%), diabetes (21.44%), hyperlipidemia (45.20%), renal diseases (24.23%), atrial fibrillation (39.25%), heart failure (26.83%) and myocardial ischemia (36.22%). Most of patients presented severe ALI (54.49%, 504/925) and most of the patients had at least one outflow tract below the knee, accounting for 60.59% of the patients (661/1091). A total of 38.68% of patients (422/1091) had no outflow tract at all. Comparing the patient characteristics of the open surgery and endovascular intervention groups, the proportion of males in the open surgery group was higher than that in the endovascular intervention group (73.11% vs. 54.58%, P < 0.001). There was no significant difference in the incidences of some complications, such as respiratory diseases, renal diseases, atrial fibrillation, heart failure and myocardial ischemia. However, the proportion of smokers in the open surgery group was lower than that in the endovascular intervention group (63.07 vs. 72.48%, P = 0.010). The proportions of patients with diabetes (27.84 vs. 20.33%, P = 0.024), hypertension (79.57 vs. 73.03%, P = 0.046) and hyperlipidemia (51.74 vs. 42.49%, P = 0.025) in the open surgery group were significantly higher than those in the endovascular intervention group. The ratio of mild ALI cases was significantly higher in the endovascular intervention group than that in the open surgery group (56.51 vs 38.82%, P < 0.001). In addition, there was no significant difference in the outflow tract below the knee between the two groups (P > 0.05). The percentages of patients with outflow tracts were 53.96% and 52.10%, respectively.

### Procedure related technical data in included patients

The overall technical success rate was 90.73% (988/1089). There was no significant difference between the open surgery and endovascular intervention groups (91.71 vs. 90.80%, P = 0.668). The open surgery procedures consisted of PAA resection with in situ graft replacement or PAA exclusion with graft bypass surgery, of which the latter was the most common procedure, accounting for 78.09% (Table 3). Vein grafts were more preferred than artificial blood vessels and accounted for 66.50% of cases. Endovascular intervention procedures mainly included catheter thrombolysis/thrombectomy and covered stent graft implantation (thrombolysis was performed preoperatively or intraoperatively in some cases), with ratios of 1.58% (5/316) and 98.42% (311/316), respectively. The covered stent grafts mentioned in all articles were Hemobahn and Viabahn covered stent grafts, and the artificial blood vessel was poly tetra fluoroethylene (PTFE).

**Table 3:** Comparisons between different open surgery or endovascular intervention procedures

Procedures	Events	Total	Percentage (%)
Open surgery			
Venous graft	544	828	66.50
PTFE graft	274	818	33.50
In situ replacement	181	826	21.91
Bypass procedure	645	826	78.09
Endovascular intervention			
Stent placement/Stent placement + thrombolysis	311	316	98.42
Thrombolysis/thrombectomy	5	316	1.58

*PTFE* poly tetra fluoroethylene

### Follow-up data

**Fig. 3:**
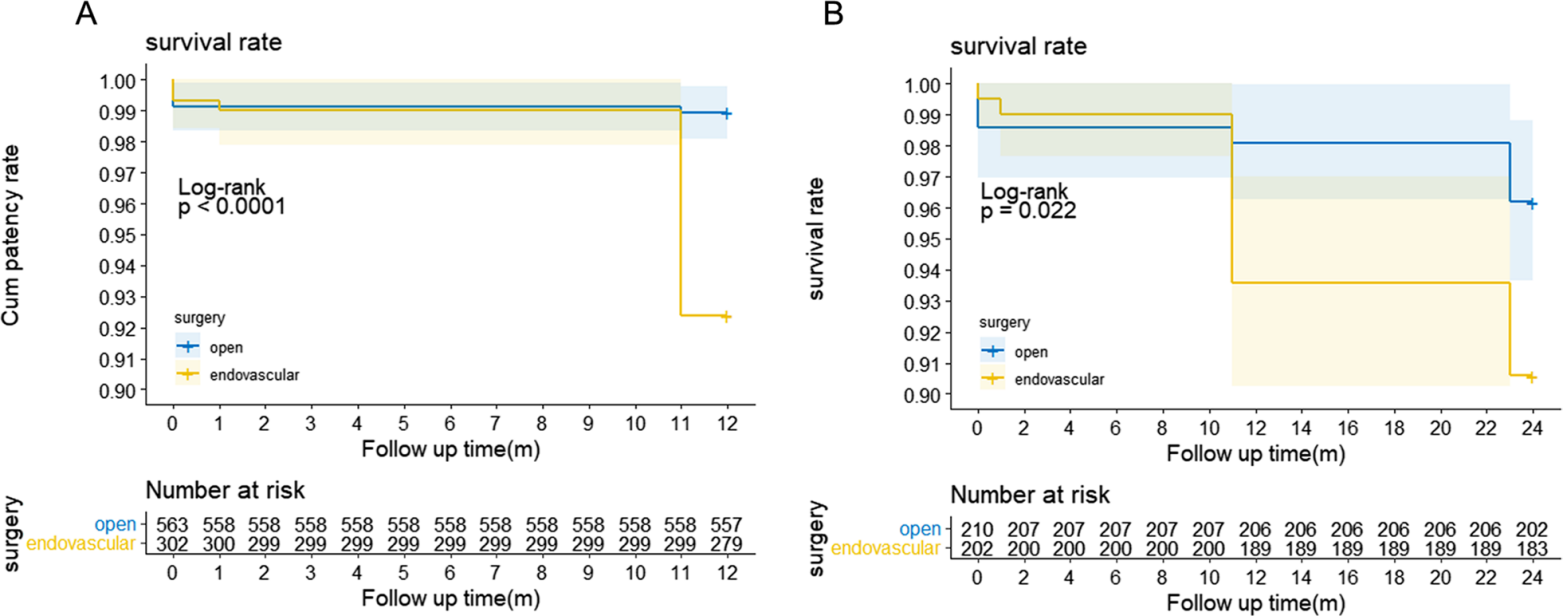
Kaplan–Meier survival analysis showed the differences in one-year **A** and two-year **B** survival rates in the two subgroups, open surgery and endovascular intervention

The total number of follow-up cases was 1083, accounting for 97.92% (1083/1106). The follow-up period ranged from 1–183 months. There was no significant difference in the rate of follow-up between the open surgery group (521/535) and the endovascular intervention group (298/305) (97.38 vs. 97.70%, P = 0.774) (Table 2). After a 1-year follow-up, the primary patency rate of the open surgery group was significantly lower than that of the endovascular intervention group (72.65% vs. 81.46%, P = 0.004), but there was no significant difference in the secondary patency rate (86.19 vs. 86.86%, P = 0.825) (Table 4). The limb salvage rate of the open surgery group was also significantly lower than that of the endovascular intervention group (83.07 vs. 98.25%, P < 0.001). During the 2-year follow-up, the primary patency rate of the open surgery group was still significantly lower than that of the endovascular intervention group (48.57% vs. 59.90%, P = 0.021), but there was no significant difference in limb salvage rate between the two groups (92.86 vs. 96.53%, P = 0.125). The 1-year survival rates were 98.93% (557/563) in the open surgery group and 92.38% (279/302) in the endovascular intervention group. The 2-year survival rates were 96.19% (202/210) in the open surgery group and 90.59% (183/202) in the endovascular intervention group. According to the Kaplan–Meier survival curve, the survival rates were significantly higher in the open surgery group both at the 1-year (P < 0.001) and the 2-year follow-up (P = 0.022) (Fig. 3). In addition, postoperative death included popliteal artery-related and nonpopliteal artery-related death. There were more nonpopliteal artery-related deaths in the endovascular intervention group both at the 1-year (P < 0.001) and the 2-year follow-up (P = 0.028) (Table 2).

**Table 4:** 1-year and 2-year follow-up data of patients in the open surgery group and the endovascular intervention group

Follow up	Variable	Open surgery	Endovascular intervention	P value
1-year	Primary patency	72.65% (409/563)	81.46% (246/302)	0.004
	Secondary patency	86.19% (206/239)	86.86% (238/274)	0.825
	Survival rate	98.93% (557/563)	92.38% (279/302)	0.000
	Limb salvage rate	83.07% (490/563)	98.25% (281/286)	0.000
2-year	Primary patency	48.57% (102/210)	59.90% (121/202)	0.021
	Secondary patency	82.74% (139/168)	85.34% (163/191)	0.501
	Survival rate	96.19% (202/210)	90.59% (183/202)	0.028
	Limb salvage rate	92.86% (195/210)	96.53% (195/202)	0.125

## Discussion

Although the morbidity of PAA was relatively low, whether open surgery or endovascular intervention is suitable for PAA has been discussed in many articles with highly reliable results [37, 38]. However, it is still unclear whether open surgery or endovascular intervention is more efficient in the treatment of thrombotic PAA with ALI. There have been no strictly randomized controlled studies exploring the best surgical treatment for this disease. The present studies on thrombotic PAA with ALI were mainly case reports with heterogeneous results [25, 30]. Marko Dragas et al. [6] reported a group of 156 patients with acute thrombotic PAA and suggested that intraoperative thrombolysis can improve the long-term patency rate and reduce the incidence of complications. The existing studies of nonthrombotic PAA found that the incidence of stent graft thrombosis and related complications among patients with stent graft implantation were higher than those associated with open surgery, and the short-term patency rate was lower, while the midterm patency rate was similar between the two groups [37]. For patients with thrombotic PAA with ALI, the complexity of the disease and the difficulty of the procedure were significantly increased, so the uncertainty of the surgical procedure options and therapeutic efficiency was also an important problem. This study compared the technical details and therapeutic efficiency of different surgical procedures by summarizing and analyzing the recent literature about thrombotic PAA with ALI.

In terms of patient characteristics, most patients had comorbidities such as hypertension and hyperlipidemia, which was similar to those described in recent studies [6]. The baseline characteristics of the patients included in the study demonstrated some differences, especially in the prevalence rates of diabetes, hypertension, and hyperlipidemia, which were higher in the open surgery group. However, there was no significant difference in the diseases that may affect the risk of preoperative anesthesia and operation, such as respiratory diseases, renal diseases and heart diseases (including heart failure and coronary heart disease). Regarding the preservation of the outflow tract below the popliteal artery, which was considered a key variable affecting the acute symptoms and therapeutic effect, there was no significant difference between the two groups. Therefore, the potential influencing factors of surgical decisions were similar between the two groups.

Based on the severity of the disease, the size and location of PAA, thrombus load and the patency of distal outflow tract, the surgery that can be selected is as follows: (1) Open surgery—PAA resection with in situ graft replacement or PAA exclusion with graft bypass surgery, combined with preprocedure (or intraprocedure) catheter thrombolysis or thrombectomy; (2) Endovascular intervention—PAA exclusion by covered stent graft combined with preprocedure (or intraprocedure) catheter thrombolysis or thrombectomy and thrombolysis/thrombectomy only. Studies have confirmed that preoperative and intraoperative thrombolysis can improve the 1-year primary patency rate [39] and limb salvage rate [40]. Preoperative and intraoperative thrombolysis can keep the arteries below the knee (such as tibial and peroneal arteries) unobstructed and maintain the distal blood flow of the lower limbs, which are helpful for long-term patency after graft bypass establishment or in situ graft replacement [41]. If acute ischemic lesions presented all distal outflow tract obstruction, the efficiency of covered stent graft implantation would be poor, and the possibility of postoperative thrombosis and in-stent reocclusion would be elevated. Therefore, it was strongly suggested that combining preoperative and intraoperative thrombolysis or thrombectomy would improve the long-term patency rate. In addition, preoperative thrombolysis can decrease the urgency of emergency surgery so that doctors will have enough time to make a more appropriate surgical plan, which will further improve the prognosis of patients [41]. Therefore, for graft bypass surgery, in situ graft replacement surgery, and endovascular intervention with covered stent graft implantation, intraoperative thrombolysis and thrombectomy affected the postoperative patency rate. However, studies comparing the therapeutic efficiency of open surgery and endovascular intervention for thrombotic PAA with ALI after thrombolysis or thrombectomy are rare. Axel Wrede et al. [33] compared the therapeutic effect of open and endovascular intervention in patients with PAA-related ALI, including 20 cases of open surgery and 19 cases of endovascular intervention. However, there was no significant difference between the two groups in the primary or secondary patency rates within the 30-day, 1-year and 2-year follow-up postoperative periods. Considering the small number of cases in this study, the results still need to be further verified.

Our systematic review included data from 1 year of follow-up for 865 limbs, including 563 limbs in the open surgery group and 302 limbs in the endovascular intervention group. During the 2-year follow-up, a total of 412 limbs, including 210 limbs in the open surgery group and 202 limbs in the endovascular intervention group, were included. Therefore, the results were reliable and representative. The results showed that the primary patency rate of the open surgery group was significantly lower than that of the endovascular intervention group, but there was no significant difference between the two groups in the secondary patency rate. The limb salvage rate of the open surgery group was also significantly lower than that of the endovascular intervention group. During the 2-year follow-up, the primary patency rate of the open surgery group was still significantly lower than that of the endovascular intervention group, but there was no significant difference in limb salvage rate between the two groups. Previous studies have confirmed that good runoff is an independent predictor of the primary patency rate for patients with endovascular intervention [42]. In our study, most of the patients who underwent endovascular intervention had preoperative or intraoperative thrombolysis, which ensured distal blood flow, so the primary patency rate of the endovascular intervention group was higher. In addition, when the patency of the distal outflow tract was equivalent, the accurate matching of the distal anastomotic artery diameter with the graft’s diameter was very important for long-term patency maintenance in the reconstructed blood vessel in the context of graft bypass surgery or in situ graft replacement. Distal anastomotic stenosis contributed to local thrombosis. Therefore, it was necessary to perform the intervention again to overcome restenosis or occlusion, resulting in a lower primary patency rate and no significant difference in the secondary patency rate. However, patients with covered stent graft implantation generally did not have the problem of graft diameter matching, and anticoagulant treatment was generally recommended after the operation. Therefore, the stent graft remained patent for a long time with good midterm therapeutic effect. In addition, the stent graft was prone to fracture, distortion or even damaged blood vessels due to mechanical stress of the knee joint movement [43]. Although the primary patency rate of the endovascular intervention group was better than that of the open surgery group within 2 years after surgery, the incidence of long-term complications increased with the extension of follow-up time, which may result in a decline in the long-term patency rate [44]. Therefore, as time went on, the difference in patency rate between the two groups still needed to be further explored. The survival rate of the open surgery group was better than that of the endovascular intervention group, and the reason may be that the average age of the patients in the endovascular intervention group was older and the possibility of suffering from other lethal diseases was higher. According to the mortality rate, the number of popliteal artery-related deaths in the two groups was similar, but the number of nonpopliteal artery-related deaths in the endovascular intervention group was significantly higher. Therefore, the survival rate of the open surgery group was higher.

## Limitation

Most of the cases included in this study were reported by a single center, and the number of cases was uneven, with differences in surgical techniques and abilities. The severity and lesions of the included cases were also diverse. Therefore, the conclusions still need to be further verified by strict randomized controlled studies.

## Conclusion

Patients with thrombotic PAA with ALI can be effectively treated by open or endovascular intervention combining preoperative or intraoperative thrombolysis with high technical success. Open surgery was dominated by venous bypass, while endovascular intervention was dominated by covered stent graft implantation. The 1-year and 2-year primary patency rates of the endovascular intervention group were significantly higher than those of the open surgery group, and there were no significant differences in secondary patency rates. The 1-year limb salvage rate of the endovascular intervention group was better than that of the open surgery group, but the 1-year and 2-year survival rates of the open surgery group were higher. However, the results of this study still need to be further verified through strict randomized controlled studies.

## Data Availability

All data generated or analyzed during this study are included in this article and are available from the corresponding author on reasonable request.

## Abbreviations

PAA: Popliteal artery aneurysm
ALI: Acute lower limb ischemia
COPD: Chronic obstructive pulmonary disease
CTA: Computed tomography of the artery
PTFE: Poly tetra fluoroethylene
